# Subchromosome-Scale Nuclear and Complete Mitochondrial Genome Characteristics of *Morchella crassipes*

**DOI:** 10.3390/ijms21020483

**Published:** 2020-01-12

**Authors:** Wei Liu, Yingli Cai, Qianqian Zhang, Fang Shu, Lianfu Chen, Xiaolong Ma, Yinbing Bian

**Affiliations:** 1 Institute of Applied Mycology, Plant Science and Technology College, Huazhong Agricultural University, Wuhan 430070, China; zhenpingliuwei@163.com (W.L.); qzhang196@163.com (Q.Z.); fangshufs@webmail.hzau.edu.cn (F.S.); chenllianfu@foxmail.com (L.C.); 2 Institute of Vegetable, Wuhan Academy of Agricultural Sciences, Wuhan 430070, China; loveylcai@163.com (Y.C.); mxl310@163.com (X.M.)

**Keywords:** *Morchella crassipes*, mating type, nuclear genome, heterothallism, mitochondrial genome, mitochondrial ORF

## Abstract

*Morchella crassipes* (Vent.) Pers., a typical yellow morel species with high economic value, is mainly distributed in the low altitude plains of Eurasia. However, rare research has been performed on its genomics and polarity, thus limiting its research and development. Here, we reported a fine physical map of the nuclear genome at the subchromosomal-scale and the complete mitochondrial genome of *M. crassipes*. The complete size of the nuclear genome was 56.7 Mb, and 23 scaffolds were assembled, with eight of them being complete chromosomes. A total of 11,565 encoding proteins were predicted. The divergence time analysis showed that *M. crassipes* representing yellow morels differentiated with black morels at ~33.98 Mya (million years), with 150 gene families contracted and expanded in *M. crassipes* versus the two black morels (*M. snyderi* and *M. importuna*). Furthermore, 409 CAZYme genes were annotated in *M. crassipes*, containing almost all plant cell wall degrading enzymes compared with the mycorrhizal fungi (truffles). Genomic annotation of mating type loci and amplification of the mating genes in the monospore population was conducted, the results indicated that *M. crassipes* is a heterothallic fungus. Additionally, a complete circular mitochondrial genome of *M. crassipes* was assembled, the size reached as large as 531,195 bp. It can be observed that the strikingly large size was the biggest up till now, coupled with 14 core conserved mitochondrial protein-coding genes, two rRNAs, 31 tRNAs, 51 introns, and 412 ncORFs. The total length of intron sequences accounted for 53.67% of the mitochondrial genome, with 19 introns having a length over 5 kb. Particularly, 221 of 412 ncORFs were distributed within 51 introns, and the total length of the ncORFs sequence accounted for 40.83% of the mitochondrial genome, and 297 ncORFs had expression activity in the mycelium stage, suggesting their potential functions in *M. crassipes*. Meanwhile, there was a high degree of repetition (51.31%) in the mitochondria of *M. crassipes*. Thus, the large number of introns, ncORFs and internal repeat sequences may contribute jointly to the largest fungal mitochondrial genome to date. The fine physical maps of nuclear genome and mitochondrial genome obtained in this study will open a new door for better understanding of the mysterious species of *M. crassipes*.

## 1. Introduction

True morels (*Morchella* spp.), an important edible mushroom distributed all over the world, especially in the North temperate zone, belong to *Ascomycota* phylum and Pezizomycetes class [1,2]. *Morchella* genus contains numerous species, with more than 334 species being recorded in Index Fungorum (http://www.indexfungorum.org, up to 10 July 2019). Early classification divided *Morchella* spp. mainly into three groups, yellow, black and red, which were also supported by the latest molecular phylogenetic data, described as Esculenta, Elata and Rufobrunnea clade [3]. Wild morels can produce ascocarps under suitable conditions in a short period of time from late spring to early summer, and their unique fragrance and taste attract a large number of hunters. For instance, in North America, many morel hunting activities were organized spontaneously in morel fruiting seasons, where participants enjoy the pleasure of hunting morels in nature, cooking and tasting them instantly [2,4]. In Europe, morels were regarded as a high-end cuisine and often appeared at important banquets. Meanwhile, the medicinal efficacy of morels was recorded in China about 600 years ago [1]. Modern medical research has shown that morel possesses various physiologically active properties, such as anti-oxidative, anti-inflammatory, anti-microbial, immunostimulatory, anti-tumor and so on [5,6,7].

Due to its delicious taste, functionality and limited amount, the market price of wild morel has reached as high as about $300 (dried) per kilogram. Therefore, many scientists have tried to domesticate and cultivate it, but failed [1]. It was not until 1982 that R.D. Ower realized the indoor cultivation of *M. rufobrunnea* (red species), but failed to put it into valuable commercial application due to the lack of basic biological knowledge and other unknown reasons [1,2,8]. On the basis of Ower’s study, China’s morel cultivation has achieved significant breakthrough since 2012 in three black species *M. importuna*, *M. sextelata* and *M. septimelata*, with an area of 9000 hectares in 2018 [9,10]. The new cultivation model was gradually introduced to France, Turkey, Australia, and the United States for small-scale trial cultivation. However, the morel cultivation industry is still facing many dilemmas due to the unsolved puzzles, and there are no evident reports about successful cultivation cases of *M. crassipes* or other yellow morels by using either the newly field cultivation model or the Ower’s indoor method [1].

Currently, the development of a cultivation technique highlights the importance of genetics research, which has enlightened a new direction to solve the problem of industrial cultivation of morels. By observing the nuclear karyotype of asci and ascospores of *M. importuna*, we have pointed out that the ascospores of *M. importuna* were multinuclear homokaryons [11]. Then, the whole genome of two monospores with different mating types was sequenced and analyzed [12], and combined with the cultivation experiments of monospores and mixed strains, we elucidated the heterothallic life cycle of *M. importuna* [13], which requires the interaction of different homokaryons representing two opposite mating types to complete the whole life history. This heterothallic characteristic has also been confirmed by other scholars through mating type gene analysis [14,15]. *M. crassipes*, like *M. importuna*, contains eight ellipsoid multinuclear ascospores; however, whether these ascospores are homokaryons is still unknown. 

*M. crassipes* (Vent.) Pers. is a typical yellow morel species with a yellow cap, honeycomb-like surface, spherical or oval shape, white stalk, and swollen foot. It naturally distributes in Europe and Asia and mainly occurs in low-altitude plain areas [16,17]. In China, it is abundantly fruiting in forests, rivers and lakes in Central Plains in early April every year [17]. Different *Morchella* species vary obviously in their physiological habits. Buscot et al. (1987, 1990, 1993) pointed out that *M. rotunda*, *M. semilibera* and *M. elata* could form an ectomycorrhizal structure with some plants [18,19,20]. Later, the ectomycorrhizal relationship between *M. esculenta* and *Pesca abies* was established by indoor culture [20]. Meanwhile, the mycorrhizal structure formed by *M. rotunda* and *P. abies* was recorded in detail by microscopic observation [19]. Dahlstrom et al. (2000) also proposed that *M. esculenta* and *M. elata* could form a typical sheath and Hattie-net mycorrhizal structure between four pine species, including *Larix occidentalis*, *Pinus contorta*, *P. ponderosa,* and *Pseudotsuga menziesii* [21]. Based on stable isotopes of carbon and nitrogen metabolism, Hobbie et al. (2016) presented that some species of *Morchella* (undetermined species) were mycorrhizal, while *M. elata* was saprophytic, with some *Morchella* samples collected from post-fire environments being the standard saprophytic fungi with an average carbon assimilation life of 11 ± 6 years [22]. Unlike other mycorrhizal fungi, *Morchella* can easily grow on various simple synthetic media, which is one of the reasons why more scientists believed their saprophytic characteristic [1,23]. The three black morel species widely cultivated in China are definite as saprophytic varieties, which do not need any living plants in the cultivation process [1]. Whether *M. crassipes* has different nutritional metabolic patterns or physiological needs as cultivable varieties is still vague.

Genome sequencing analysis promotes a further understanding of the polarity, life cycle and nutritional ecotypes of fungi, such as *Tuber melanosporum* and *Laccaria bicolor* [24,25]. Currently, the genomic sequences of *M. importuna* and *M. septimelata* in the family Morchellaceae are published, and 1k genome projects have sequenced *M. snyderi*, *M. septimelata* and *M. conica*, all of which belong to the black group [12,26]. Mitochondria, typically referred to as the power factories of cells due to their contribution to energy supply, also participate in ion homeostasis, intermediary metabolism and cell senescence and apoptosis [27,28]. Compared with other fungi, the mitochondria of *M. importuna* have distinct characteristics in genome size, ncORF (non-conserved open reading frames), intron, and repeat sequence, with its mitochondrial genome once being regarded as the largest in size [29]. However, whether the mitochondrial genome of *M. crassipes* also has the same characteristics is not known, and a comparative analysis of the mitochondria of neighboring species will help to understand their evolution. In this paper, we reported the subchromosome-scale genome physical map and the complete mitochondrial genome of *M. crassipes*. Additionally, the characteristics of the nuclear genome and mitochondrial genome of *M. crassipes* were also analyzed.

## 2. Results

### 2.1. Genomic De Novo Assembly

For the first time, three-generation long fragment sequencing and two mate-pair Illmuia jumping sequencing were used to *de novo* assemble the genomes of *M. crassipes,* resulting in a 56.7 Mb genome with the longest sequence length of 7.34 Mb. The number of contigs and scaffolds was 97 and 23, and the size of N50 was 814.08 kb and 3.00 Mb, respectively. Based on the estimated genome size (58.64 Mb) of the M10M26 strain by kmer spectrum analysis, 96.7% of the genome (56.7 Mb) was obtained (Table 1). The assembled scaffolds were checked by eukaryotic conserved telomere structure (TTAGGG/CCCTAA) [12]. Telomere structure was detected in most scaffolds at one or both ends, except for three scaffolds (Morcra10GS4, Morcra10GS8 and Morcra10GS19) without telomere structure. At both end of 8 scaffolds (LG1 to LG8), complementary telomere structures were identified with the repetitive unit of TTAGGGTTAG or CTAACCCTAA, implying that they might be complete chromosomes. According to complementarity, the chromosome number of *M. crassipes* was at least 14. The BUSCO genome completeness evaluation results illustrated that 98.62% of the genome integrity was obtained. The final *de novo* genomic assembly results were submitted to NCBI, with the public accession number of WBVU00000000.

### 2.2. Genomic Structure and Characteristic Analysis

The total number of DNA and RNA transposon elements (TEs) was 2139, with a whole length of 3,349,586 bp, accounting for 5.90% of the genome size. Nine DNA TEs were detected, mainly including hAT-Ac and CMC-EnSpm with numbers of 182 and 117, accounting for 0.44% and 1.09% of the length of the TEs, respectively. Gypsy (LTR) and Tad1 (LINE) were the main RNA TEs, with numbers of 1276 and 178, covering 29.30% and 3.73% of the length of the TEs, respectively. Additionally, more repeat-like consensus sequences were found unable to be reliably classified, which occupied 9.90% of the genome. Therefore, a total of 8,703,914 bp sites, about 15.34% of the genome, were identified to be repeats in the genome of *M. crassipes* (Table S1).

Simple sequence repeat (SSR) analysis detected 28,097 SSRs, with a total length of 565,583 bp. In addition to single base repetition (19,690), triple base repetition (3922) was the main type, followed by dibasic (3295) and tetrabasic (729) repetition. Considering sequence complementary, A/T, AG/CT and ACC/GGT were the most frequent repeats among monobasic, dibasic and tribasic tandem repeats.

### 2.3. Protein Prediction and Functional Annotation

According to the alignment of homologous proteins and the RNA-seq data and *ab initio* prediction, a total of 11,565 protein-encoding genes were predicted. The genomic structure analysis revealed that the gene median length was 1639 bp, intergenic median length was 1603 bp, cDNA median length was 1460 bp, average exon number was 3.8, and the single-intron median length was 63 bp. Of the protein-encoding genes, 96.44% (11,154/11,565) were annotated using the Nr (NCBI non-redundant database, 92.5%, 10,699/11,565) and InterPro (94.9%, 10,976/11,565). The species distribution of the Nr top hit showed the highest protein-number similarity of *M. crassipes* with *M. conica* (9901), followed by *Choiromyces venosus* (7009), *T. borchii* (6642), and *T. aestivum* (6376), which clearly demonstrated the lack of studies on closely related species of *M. crassipes*.

In the whole genome of *M. crassipes*, a total of 409 CAZYme genes were identified, including 196 glycoside hydrolases (GH), 82 glycosyltransferases (CT), 22 polysaccharide lyases (PL), 26 carbohydrate lipases (CE), 42 carbohydrate domains (CMB), and 77 auxiliary enzymes (AA) (Figure S1). Compared with *M. importuna* and *M. snyderi*, *M. crassipes* was smaller in the gene number of CE (26/29/29) and CMB (42/47/46), but larger in number of the other enzymes GH (196/181/175), GT (82/70/72), PL (22/22/21), and AA (77/70/68).

Furthermore, similar to *M. importuna,* 309 transcription factors of 29 different types were examined in *M. crassipes* (Table S2) [12]. Consistent with other filamentous ascomycetes, Zn2Cys6 was ranked first in the number of transcription factors (*n* = 99), followed by homeodomain-like (*n* = 61) and Zinc (*n* = 50), respectively.

### 2.4. Phylogenetic Tree Construction and Evolution Analysis

**Figure 1:**
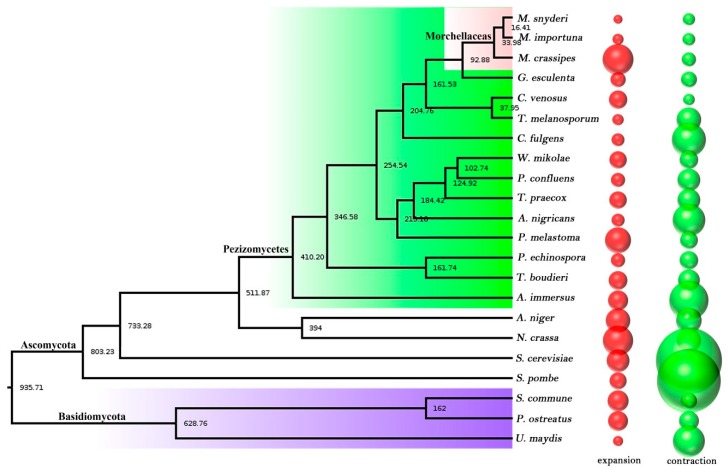
The phylogenetic trees and gene family comparative analysis of 22 species. The number of phylogenetic tree nodes indicated the divergence time, Mya (million years); the bubble diagram on the right represented the expansion (red) and contraction (green) of the gene family among species; and the diameter of the bubble represented the number of genes that expanded and contracted.

Together with two open *Morchella* species (*M. snyderi* and *M. importuna*) and 19 other fungal genomes, a phylogenetic tree was constructed by using the maximum likelihood method and LG+I+G+F model with 894 single-copy proteins identified in the orthology analysis. Based on the fossil calibration point, the divergence time of each species could also be calculated. All branch nodes of the phylogenetic tree constructed were 100%, and the topological structure was consistent with the early classification studies (Figure 1) [30]. Three morel species were clustered in one small branch, and two black morels *M. snyderi* and *M. importuna* had the closest genetic relationship with the differentiation time of about 16.41 Mya. As a yellow morel species, *M. crassipes* had a slight genetic distance from the two black morels and differentiated at about 33.98 Mya. Morchellaceae (three morels) was differentiated with Discinaceae (*Gyromitra esculenta*) at the family level at about 92.88 Mya, and with Tuberaceae (*C. venosus* and *T. melanosporum*) about 204.76 Mya, which was consistent with previous studies [12].

### 2.5. Contraction and Expansion of Gene Families 

There was no significant number difference between the two black morel species in terms of gene family contraction and expansion. At the *p* < 0.05 level, 110 gene families contracted (71) or expanded (39) in *M. snyderi*, and 111 gene families contracted (68) or expanded (43) in *M. importuna*. However, there was a distinct difference in *M. crassipes*, with 150 gene families contracted or expanded. Specifically, 65 gene families (160 genes) were contracted just like the case in the two black morel species, but 85 gene families (738 genes) were expanded, far more than the number in *M. snyderi* and *M. importuna* (Figure 1). Species vary in their types of gene family contraction and expansion. In *M. crassipes*, COG295 (18/7, *p* = 0), COG238 (16/5, *p* = 0) and COG4953 (9/1, *p* = 0) were the most prominent expanded gene families. Gene annotation analysis showed that they represented ribonuclease H-like domain, transposase domain-containing protein and jmjC domain-containing demethylation protein 1, respectively. The most obvious contracted gene families were COG33 (1/9, *p* = 0) and COG15 (2/7, *p* = 0), which were also expanded significantly in the two black morel strains, but without known functional annotation, which might play a special role at the species level in morels.

Based on the enrichment results of GO and KAAS in these extended and contracted gene families of *M. crassipes*, the expanded COG genes were mainly enriched in DNA integration (6.2 × 10^−34^), retrotransposon nucleocapsid (6.8 × 10^−23^), hydrolase activity on glycosyl bonds (2.1 × 10^−16^), DNA metabolic process (4.1 × 10^−16^), and 22 other special functions at the GO level. At the KAAS pathway metabolism level, the basic metabolic processes were mainly enriched, such as biosynthesis of other secondary metabolites (2.0 × 10^−20^), metabolism of other amino acids (4.8 × 10^−16^), carbohydrate metabolism (2.1 × 10^−12^), and another five basic metabolism pathways and one spliceosome pathway. In the functional analysis of the contractile genes, the genes in *M. importuna* were used for enrichment analysis, which showed that at the GO level, the contractile genes were enriched in the two categories of telomere maintenance (6.08 × 10^−5^) and telomere organization (1.12 × 10^−4^), but not in the KAAS pathway.

### 2.6. Mitochondrial Genome Assembly and Annotation of M. crassipes

A gradual extension assembly strategy was used to obtain the 531,195 bp circular mitochondrial physical map of *M. crassipes* with a GC content of 46.2% (GenBank accession number: MN542893). A total of 14 core conserved mitochondrial protein-coding genes, 2 rRNA and 31 different tRNAs were identified to be widely distributed in the mitochondrial genome of *M. crassipes* and on the sense strand (Figure S2). Additionally, 412 mitochondrial ncORFs in the length range of 300 to 2460 bp (orf819) were predicted, with 231 of them on the sense strand, and the remaining 181 on the antisense strand. If the interval regions of the two rRNA (*rrnS* and *rrnL*) genes were included, 51 mitochondrial introns were determined, which were *nad*1 (2), *cob* (4), *nad*2 (3), *nad*4 (1), *cox*1 (6), *cox*3 (4), *cox*2 (3), *nad*5 (9), *rrnS* (6), and *rrnL* (13), respectively. These 51 introns spanned 285,118 bp, accounting for 53.67% of the whole mitochondrial genome. It is notable that 19 of them exceeded 5 kb in size, with the maximum intron length being 36,257 bp (cox1i1), followed by 27,074 bp (cox1i4) and 14,501 bp (cox1i6), all of which appeared in *cox*1 gene (Figure S2, Table S4). These large intron regions also led to difficulties in gene prediction, and in these regions, 221 ncORFs were distributed, with 133 and 88 of them being consistent with the typical mitochondrial genes on the sense strand and the antisense strand, respectively. A total of 12 intron structures were identified by RNAweasel, including 51 group I introns and 16 group II introns, and except for two group II (domainV) introns and one group IC1 intron, all others were located in the introns of typical mitochondrial genes. On the other hand, 41 of the 51 introns were identified to have intron structural fragments by RNAweasel (Table S4). Although nearly 70% of the ncORFs could be annotated by Nr or InterPro databases, most of them were hypothetical proteins except for 75 homing endonuclease (LAGLIDADG endonuclease or GIY-YIG endonuclease) genes and 14 typical mitochondrial conserved genes. Interestingly, 61 of these homing endonucleases occurred in the intron region of the conserved gene. 

### 2.7. Mitochondrial Genome Characteristic Analysis of M. crassipes 

The large number of mitochondrial ncORFs was a very interesting feature of *M. crassipes*, and their total length (412 ncORFs) reached 216,906 bp, accounting for 40.83% of the whole mitochondrial genome. However, the sequence length of all tRNA, rRNA and mitochondrial conservative genes was just 22,073 bp, accounting for 4.15% of the mitochondrial genome. 

The RNA-seq data at the mycelial and sclerotial stages were compared with the mitochondrial genome by Hisat2, so that the expression of each mitochondrial gene or ncORF could be calculated. The results showed that except for *rps3*, mitochondrial conservative genes and the two rRNA genes had definite expression activity, and with expression activity being found in 297 of the 421 ncORFs. The expression level was highest for *orf*167 (TPM = 162,130.585), followed by *rrnL* (TPM = 141,294.319), *orf*109_6 (TPM = 93,078.770), *rrnS* (TPM = 63,204.311), *atp*8_2 (TPM = 50,107.772), and *orf819* (TPM = 49,978.890). In Nr annotation, the *orf*819 was annotated as a reverse transcriptase (*e* value = 3.88 × 10^−128^) and had 86.06% similarity (*e* value = 0) with the *orf*771 of *M. importuna*, both of which were highly expressed in the mitochondrial genome and should have the same unknown physiological function. The *orf*167 and *orf*109_6 failed to be annotated in the NR database, and the Interpro annotation revealed that *orf*167 contains a domain as the region of a membrane-bound protein predicted to be outside the membrane, which should be mitochondrially encoded and secreted to mitochondria for action in vitro; however, the *orf*109_6 still failed to be annotated in any database.

**Figure 2:**
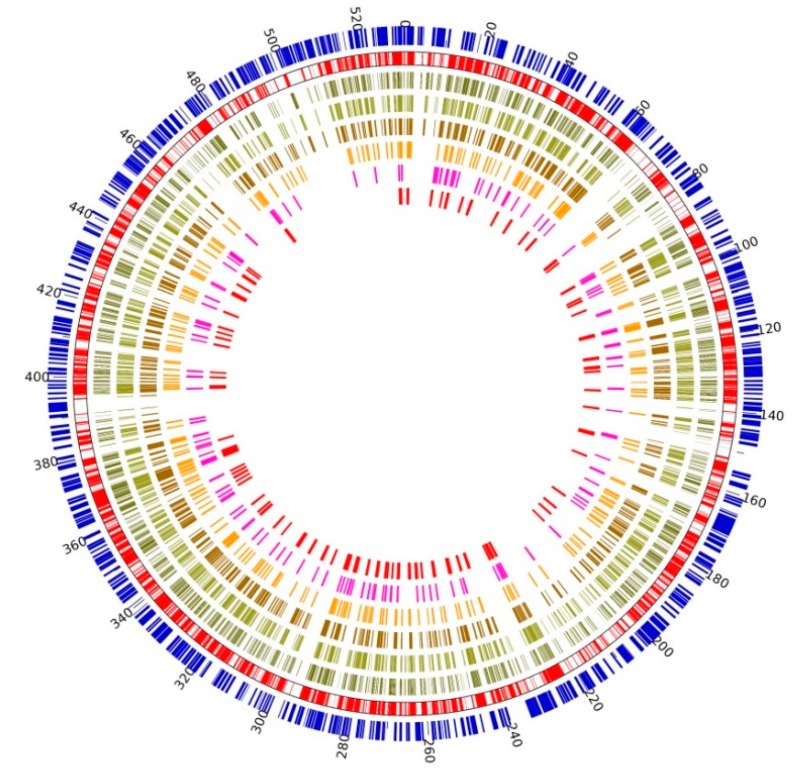
Self-repetition in the mitochondrial genome of *M. crassipes.*From the inner circle to the outer circle, the six histograms in the inner circle showed the position and length of repetitive sequences with different lengths (red histograms, repeat sequence length > 800 bp; lavender histograms, repeat sequence length between 600–800 bp; orange histograms, repeat sequence length between 400–600 bp; dark brown histograms, repeat sequence length between 200–400 bp; light brown histograms, repeat sequence length between 100–200 bp; light gray histograms, repeat sequence length less than 100 bp); the outer second red circle showed the position information overlapping all repetitive sequences; and the outer blue bars showed the position information of genes without considering the direction.

As the largest fungal mitochondrial genome so far, in addition to abundant ncORFs and intron fragments in large regions, another very obvious feature was the repetition of mitochondrial sequence fragments (Figure 2). Under the threshold of 10^−5^, a total of 13,614 independent repeats were identified in the mitochondrial genome of *M. crassipes*, including 419 repeats of fragments larger than 1000 bp, 5272 repeats of fragments in the length range of 200 bp to 1000 bp, 11,886 repeats between 100 bp to 200 bp, and 9710 repeats less than 100 bp (Figure S3). The total length of unique repeat regions was 272,544 bp, accounting for 51.31% of the whole mitochondrial genome. Location analysis of repeat units of different lengths revealed obvious hotspots of repeat regions in the mitochondrial genome. The locations of repetitive regions, especially large repetitive regions (>600 bp), had mostly occurred between mitochondrial genes and ncORFs (Figure 2 and Figure S3). BLAST (*e*-value = 10^−5^) comparison of the mitochondrial genome with the nuclear genome of *M. crassipes* showed that there is no similarity between them except for a few fragments, which had been proved to be incorrectly assembled in the nuclear genome. The percentage of GC in the whole mitochondrial genome decreased to 39.30% after deducting the sequence of repetitive regions, indicating that the increase of the mitochondrial genome of *M. crassipes* can be mainly attributed to the repetition of some high GC content fragments in the genome. This large amount of internal repetition could also be considered as one of the main reasons for the failure of conventional mitochondrial genome assembly strategies.

## 3. Discussion

### 3.1. Comparative Genome Analysis of M. crassipes

*Morchella* is a mysterious species [1,2,4]. Around the 1980s, a lot of physiological and genetic studies were performed, followed by stagnation as its complete life history is vague in the laboratory [1]. In recent years, the successful cultivation of some varieties has rekindled the study of molecular genetics, taxonomy and metabolism of *M. importuna* [9,12,31]. Genome sequencing of two different mating-type monospores of the *M. importuna* cultivated strain showed that the nuclei with different polarities played different physiological roles at genomic, genetic and functional levels [12]. Combined with cytological and cultivation experiment, the heterothallic life cycle of *M. importuna* was confirmed [11,13]. Additionally, 1k genome projects and other researchers have sequenced three other black *Morchella* species (*M. synderi*, *M. exime* = *M. septimelata*, *M.concia*) [26], all of which have been released in the JGI website and played a significant role in promoting the related scientific research. The genome of *M. crassipes* released in this paper is the first yellow strain genome currently. Generally, *M. crassipes* was slightly larger than *M. importuna* in the genome size, but they had a similar repeat sequence proportion of 15.34% and 15.20%, respectively. Meanwhile, they were different in the proportion of transposon element and non-characteristic repeat sequences: 5.90% of transposons element and 9.90% of non-characteristic repeats of *M. crassipes*, in contrast to 7.74% and 7.66% for *M. importuna*, respectively. Therefore, *M. crassipes* was also smaller in gene density, with 204 genes per Mb base. Compared with morels, the *G. esculenta* (CBS101906 v1.0) with the closest evolutionary distance had a similar genome size and gene content. However, it was obviously different from the two adjacent mycorrhizal fungi, *Choiromyces venosus* (120613-1 v1.0) and *Tuber melanosporum* (Mel28 v1.2), which had a high transposon element of 53% and 58%, respectively (Table S3) [24].

Gene family contraction and expansion analysis illustrated that *M. crassipes* and two black morels differed severely in gene types. It is apparent that 738 genes expanded in *M. crassipes*, sharply more than 62 in *M. snyderi* and 96 in *M. importuna*. The number of genes expanded in *M. crassipes* was the largest in the whole class (Figure 1), and functional enrichment analysis reflected that the main function of these expanded genes was related to DNA metabolism. Interestingly, 160 contracted genes in *M. crassipes* were enriched in telomere maintenance (6.08 × 10^−5^) and telomere organization (1.12 × 10^−4^), while those expanded genes in *M. importuna* were also enriched in telomere maintenance (2.7 × 10^−4^) and telomere organization (4.01 × 10^−11^). This indicated the potential specificity of telomere maintenance and organization in *M. impotuna*. Telomeres play an important role in maintaining the integrity of chromosomes and controlling the cell division cycle, which are important control elements for species aging and longevity [32]. The black morels *M. elata*, *M. importuna* and *M. sextelata* had developmental aging characteristics [33], and after subculture for a period of time, they would undergo an irreversible aging accompanied with autophagy, apoptosis and necrosis. The lipid peroxidation content increased dramatically, the sclerotinia-producing ability decreased, the pigments increased, and the yield decreased significantly with the aging degree increased in *M. importuna* and *M. sextelata* strains [34]. Whether this is the characteristic of different morel varieties or not and whether *M. crassipes* can eliminate severe aging or not need to be studied further.

### 3.2. Comparative Analysis of CAZYme Enzymes

There was no significant difference in CAZYme enzymes among the three *Morchella* strains, with 116, 127 and 118 for *M. crassipes*, *M. importuna* and *M. snyderi*, respectively. This was consistent with physiological data that they could grow robustly on ordinary synthetic media. The main difference was reflected in the PL24 (ulvan lyase, EC 4.2.2.-) enzyme, in which two genes were identified in *M. crassipes*, but not in the two black *Morchella* species. Besides, *M. importuna* had more CBM20 (starch-binding domains), CE2 (acetyl xylan esterase), and GT28 (EC 4.2.1.-) genes. Furthermore, the genes related to cellulose, hemicellulose, lignin, and pectinase in plant cell wall degradation were analyzed comprehensively. All in all, there were no significant differences in these enzymes among the three *Morchella* species. There were three deletions in the hemicellulase of *M. crassipes*, namely CE2 (0/1/0), CE7 (0/1/1) and GH39 (0/1/1), while the two black *Morchella* species had more similar CAZYme genes. Meanwhile, *M. crassipes* had more genes in cellulase and ligninase in contrast to more genes in pectinase for the two black *Morchella* species.

Compared with the four identified mycorrhizal fungi (*T. melanosporum*, *C. venosus*, *Wilcoxina mikolae* and *Terfezia boudieri*) and three identified saprophytic fungi (*Pyronema confluens*, *Tricharina praecox* and *Peziza echinospora*), the three *Morchella* species and one *G. esculenta* had more comprehensive and diverse plant cell wall degrading enzymes. This suggests that *G. esculenta* may have the same nutritional metabolic pathway as morels, which was classified as a false morel, with the nutritional ecotype being unknown [35]. The three *Morchella* species and *G. esculenta* were tremendously rich in cellulase, hemicellulase and pectinase prior to mycorrhizal fungi and saprophytic fungi, while approximately equal in the ligninase. Overall, the genes of plant cell wall degrading enzymes in *Morchella* covered almost all of that in mycorrhizal fungi, which means that *Morchella* may also adopt the mycorrhizal growth pattern when necessary.

### 3.3. M. crassipes Is a Heterothalic Fungus

**Figure 3:**
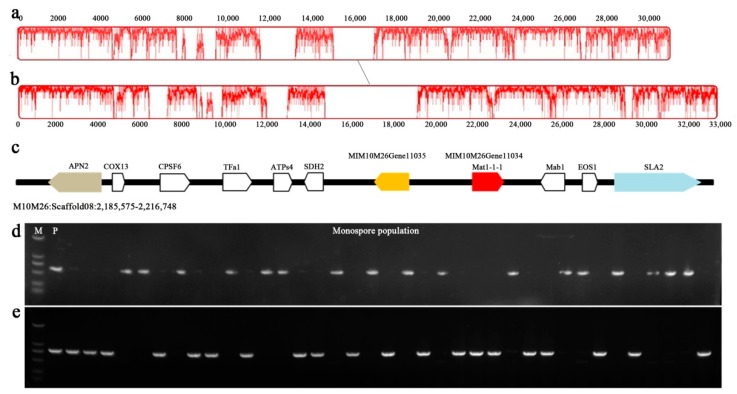
Collinearity analysis of Mat1-1 loci of *M. crassipes* and *M. importuna.* The diagonal line showed the Mat1-1 loci collinear region between *M. crassipes* (**a**) and *M. importuna* (GenBank accession number: MK527108) (**b**) by Mauve software, and the peak graph on the coordinate axis represented the degree of similarity of the collinear region. The gene order of the Mat1-1 locus of *M. crassipes* was shown (**c**). Electrophoresis maps represented the amplification of Mat1-1-1 (**d**) and Mat1-2 (**e**) genes in monosporic populations of *M. crassipes* (M, DM2000 molecular marker; P, M10 tissue isolation parent strain; 37 monosporic populations isolated from M10 ascocarp was used for mating structure detection).

In this paper, the monospore M10M26 of *M. crassipes* was sequenced in depth and assembled. The final assembly genome coverage reached 96.7% of the predicted size. Only one mating locus, scaffold08:2185575-2216748, was identified by genome-wide scanning analysis. The MAT loci of *M. crassipes* and *M. importuna* had a consistent gene sequence (Figure 3). Both ends had APN2 and SLA2 flanking genes specific to the mating boundary as other *Ascomycetes*, but the difference was obvious in base sequences [12]. There were only 61% overlapping regions between the two sequences, and the overall similarity was 85.60%. This indicated that the sequence of these two species had a higher evolutionary rate. The mating locus of *M. crassipes* was about 10 kb in length, and in this region, two opposite genes were predicted in this region, MIM10M26Gene11035 and MI10M26Gene11034. The amino acid sequence length of MIM10M26Gene11034 gene was 564aa, and 143–234aa was annotated as the conserved domain of MATalpha_HMGbox (pfam04769, 2.51 × 10^−5^), which was most similar to the *M. crassipes* mating protein in the Nr database, with 97.01% of the amino acid sequence (ALX87365.1, query cover = 99%, *e* = 0). The similarity was 62.14% (MIM04M24Gene05715, query cover = 99%, *e* value = 0) with mat1-1-1 in our previous publication [12]. MIM10M26Gene11035 had the amino acid length of 419aa, which was a hypothetical protein with no conservative domain in the Nr database. The similarity of MIM10M26Gene11035 corresponding to *M. importuna* (MIM04M24Gene05716) was 34.06% (query cover = 57%, *e* = 1 × 10^−40^). Transcription factor database annotations signified that MIM10M26Gene11035 was a transcription factor of homeobox class (IPR001356, 362-407aa, *e* value = 5.99 × 10^−7^); whether this gene reflected the mating type needs further study.

Mating type-specific primers were designed for mat1-1 according to the genome sequences of *M. crassipes*, and the mat1-2 primers were designed with the help of the preliminary sequencing of other *Morchella* genus sequencing. The results showed that only one mating type gene fragment could be amplified from each ascospore, and two MAT gene fragments were obtained from the mycelium of tissue isolation (Figure 3d,e). *M. crassipes*, like *M. importuna*, is a heterothalic fungus [12,13].

### 3.4. Comparative Analysis of Mitochondrial Genome

**Figure 4:**
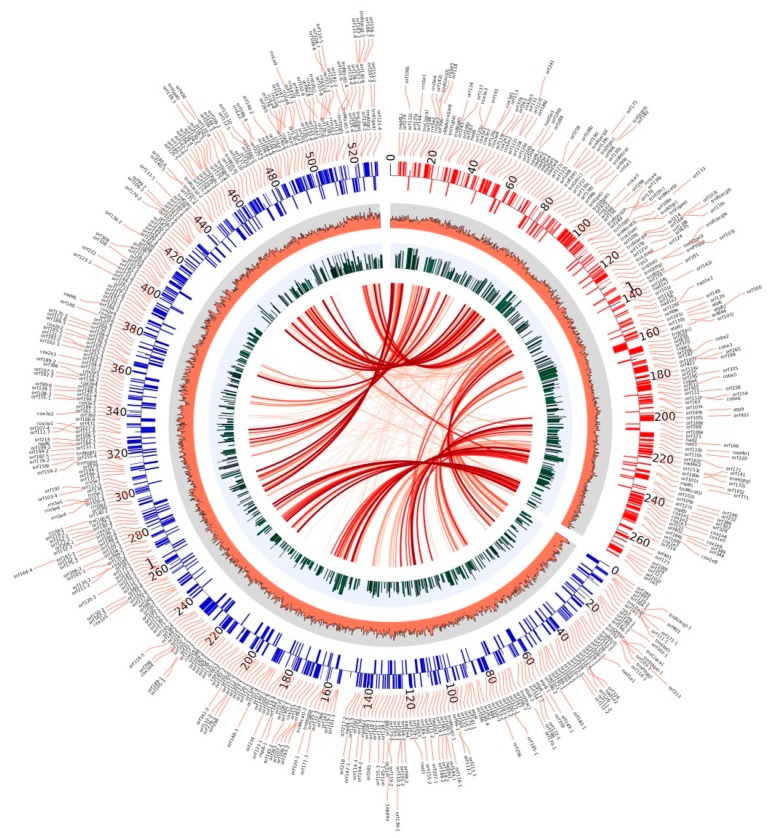
Comparative analysis of mitochondria between *M. crassipes* and *M. importuna.*On the upper right side, the mitochondrial genome of *M. importuna* with a size of 272 kb (GenBank accession number: MK527108) can been, and on the left, the mitochondrial genome of *M. crassipes* with a size of 531 kb (GenBank accession number: MN542893) can be seen. The red and blue bars of the outer track marked the location and direction of the genes (outward was the sense strand, and inward was the antisense strand), and the name of the gene was displayed in the outer circle with links; from the outside to the inside, the second circle was the GC percentage content; the third circle was the expression level of the genes in the mycelial and sclerotial stages; the inner circle was the connection of repeated sequences between *M. crassipes* and *M. importuna*, with the lines from light to deep color reflecting the length of the repeat sequence that increased.

*Morchella* species seem to have a special mitochondrial structure. The present mitochondrial genome of *M. crassipes* and that of *M. importuna* were the two largest ones of all fungi so far [29]. Except for a *nad*4L gene between *cox*1 and *nad*6 genes in the mitochondrial genome of *M. importuna*, and a truncated *nad*4_2 gene fragment between *nad*4 and *cox*1 in that of *M. crassipes*, the two species were consistent in the conservative gene sequence. The main differences were in the number and sequence of ncORFs. A total of 412 ncORFs were predicted in the mitochondrial genome of *M. crassipes*, in contrast to 151 in that of *M. importuna* (Figure 4). At the threshold of 10^−5^, 97 ncORFs of *M. crassipes* have high homology with 77 ncORFs of *M. importuna*, suggesting that they might come from the same evolutionary ancestors, but had obvious strain specificity and rapid evolutionary characteristics. Interestingly, despite notable differences in the total number, an approximate proportion of ncORFs (72.1% in *M. crassipes* and 71.5% in *M. importuna*) was expressed at vegetative growth stages, implying that those ncORFs of the two species may have some specific functions. In some fungi with larger mitochondrial genomes, the presence of ncORFs was also predicted, such as *Rhizoctonia solani* (119 ncORFs, 235 kb mitochondrial genome) [36], *Phlebia radiata* (108 ncORFs, 156 kb mitochondrial genome) [37], *Sclerotinia borealis* (80 ncORFs, 203 kb mitochondrial genome) [38], and *Ophiocordyceps sinensis* (58 ncORFs, 157 kb mitochondrial genome) [39]. However, no research has yet been conducted on the functions of these ncORFs. The large number of ncORF was one of the main reasons for the enlargement of the mitochondrial genome of *M. crassipes*.

Both the number and length of introns in the mitochondria of *M. crassipes* were the largest among all fungi to date. The sequence length of 51 introns accounted for 53.67% of the total mitochondria genome length, with a length of more than 5 kb for 19 of the 51 introns, much larger than that of other fungi, thus increasing the difficulty of predicting conservative genes. These large introns were also present in *M. importuna* and considered as a manifestation of greater mitochondrial tolerance [29]. Another distinct feature was sequence repetition within mitochondria. The repetitive sequences in *M. importuna* were about 18%, while those in *M. crassipes* were as high as 51.31%, with no definite repetitive features (no transposable features), just like *R. solani* and *M. importuna* [29,36]. A comparative analysis of the mitochondrial genome with the nuclear genome and other public databases (nr/nt, refseq_genomes, wgs, HTGS and gss) unveiled that no collinearity existed, suggesting that the main reason for the increase of the mitochondrial genome of *M. crassipes*, to some degree, came from the interior. Overall, the large number of ncORFs, high content of introns, and high degree of internal repetitive sequences all contributed to this super-large mitochondrial genome in *M. crassipes*. The comparative analysis of species with close genetic distances may help us to uncover the time and internal driving forces of the enlarged mitochondrial evolution.

## 4. Materials and Methods

### 4.1. Strain Selection and Material Preparation

*Morchella crassipes* M10, a wild yellow strain collected in the suburb of Zhengzhou in April 2008, was identified by morphological characteristics and molecular phylogenetic tree construction [17]. The specimen and the strain had been deposited in the Preservation Center of the Institute of Applied Mycology, Huazhong Agricultural University. The tissue isolation was performed in the stipe, and the monospore population was obtained by micromanipulator. Mycelia were cultured in complete yeast extract liquid medium (CYM) (glucose 20 g·L^−1^, yeast extract 2 g·L^−1^, peptone 2 g·L^−1^, K_2_HPO_4_ 1 g·L^−1^, MgSO_4_ 0.5 g·L^−1^, and KH_2_PO_4_ 0.46 g·L^−1^) at 23 °C for 7 days for DNA extraction. The M10M26 monospore strain was used for genome sequencing. M10 (mycelial and sclerotial tissues grown on CYM liquid medium for 3 days, 7 days and 14 days) was used for RNA-sequencing. The DNA and cDNA were prepared as reported by Liu et al. and Zhang et al. [12,40]. The information of the strains has been deposited in the NCBI with bioSample number SAMN12905432 (M10) and SAMN12916226 (M10M26).

### 4.2. Genome and Transcriptome Sequencing

The M10M26 monospore strain was sequenced using Illumina and PacBio sequencing scheme. Three insert fragments of 270 bp (PE150, 4G raw data, Illumina Hiseq 4000, Illumina Inc., San Diego, CA, USA) and one 800 bp (PE125, 4G raw data, Illumina Hiseq 4000) PCR-free library were selected for paired-end sequencing, and one 8 Kb and one 10 Kb mate-pair library were selected for jumping sequencing (MP50, 4G raw data, Illumina Hiseq 4000). The PacBio RSII sequencing platform (PacBio P6-C4, Menlo Park, CA, USA) was used for third-generation sequencing, and the raw data of two SMRT cells were generated from the length of the 20 Kb library. Meanwhile, the RNA-sequencing of the M10 strain was carried out with an insert fragment length 270 bp (PE150, 6G raw data, Illumina Hiseq 4000). All reads have been deposited in the SRA at NCBI, under accession numbers SRR10230725–SRR10230733.

### 4.3. Genome Assembly and Annotation

The detailed genome assembly process has been reported in our previous work [12]. Briefly, the Trimmomatic software (trimmomatic-0.33) was used to filter the raw reads, followed by using ALLPATHS-LG software (version: 44849) for *de novo* genomic assembly with the Illumina data [41], and the DBG2OLC, FALCON and Quickmerge software to mix the Illumina and PacBio data for *de novo* genomic assembly [12,42,43]. The SSPACE software (SSPACE_Standard_v3.0) was used to connect scaffold with Illumina mate-pair data [44], and GAPCLOSER software (version: v1.12) was used to fill the gaps in the assembly with the paired-end data [45].

For gene prediction, RNA-sequencing data and homologous protein comparison, *ab initio* prediction strategies were implemented. First, the RepeatMasker (v 1.332) and RepeatModeler (v 1.0.11) software were used for the prediction of repetitive sequences [46,47]. Subsequently, the Hisat2 software was used to compare the filtered RNA-seq data with the initial genome for obtaining the gene models [48]. Then, the GeneWise software (wise2-4-1) was used for homologous protein comparison [49]. A more complete genetic model for Augustus (v 3.3.2) was obtained by integrating transcriptome and homologous protein comparisons [50]. Lastly, the above predictive results were screened using PFAM database to acquire the final gene prediction results [51].

The amino acid sequences of the predicted proteins were compared with the Nr and Interpro databases using the BLAST method for gene annotation. Next, the gene ontology (GO) annotation was performed with the Blast2go software [52]. The dbCAN V6.0 software was used for CAZyme family annotation [53]. The transcription factor (TFs) family was annotated by the InterPro database [54]. Simple sequence repeat (SSR) was analyzed by MISA perl script (version 1.0) [55]. Additionally, the completeness of the genome was evaluated using BUSCO fungi_odb9 [56].

### 4.4. Mitochondrial Genome Assembly, Gene Annotation and Bioinformatics Analysis 

Due to the ineffectiveness of conventional assembly strategies in mitochondrial genome assembly caused by the large proportion of repetitive sequences, the gradual extension strategy was carried out as described in our recent work [29]. The mitochondrial ORFs were identified automatically with MFannot and RNAweasel using genetic code 4 (http://megasun.bch.umontreal.ca/RNAweasel/), and the prediction results were corrected by the homology comparison method. The tRNAs were identified by tRNAscan-SE (v 2.0.2) [57]. Functional assignments were performed by BLASTP searching Nr and InterPro databases. The transcriptional regulation of mitochondrial genes was evaluated using RNA-seq data by Hisat2 [48]. Repetitive sequences were identified and analyzed by local BLASTN searches of mtDNA against itself or nuclear genome at a cut-off *e*-value of 10^−5^. The mitochondrial genome collinearity was analyzed by Mauve [58]. The mitochondrial physical map was drawn using Circos software (v 2.30) [59].

### 4.5. Phylogenetic Tree Construction and Comparative Genomic Analysis

Phylogenetic tree construction and comparative genomic analysis were conducted among *M. crassipes*, *M. importuna* (NCBI: QOKS01000000), *M. snyderi* (JGI: DOB2424 v1.0), and another 19 fungal genomes downloaded from the JGI or NCBI public database (For details, refer to Table S3). First, the OrthoMCL v2.0.9 software was used for the orthology and paralogy analyses, the MCL v12-068 software was used for clustering of homologous genes [60], the MAFFT v7.158b software was used for multiple sequence alignment, and the conservative blocks were extracted by the Gblocks v0.91b software [61]. Then, Prottest v3.4 software was used to calculate the best amino acid replacement matrix [62], and the RAxML v8.1.24 software was applied to calculate the maximum likelihood species trees [63]. After a species tree was constructed, the dating analysis was performed using r8s v1.81 software with *Schizophyllum commune*, *Pleurotus ostreatus*, *Aspergillus niger*, and *Neurospora crassa* as a fossil calibrated point [64,65]. Finally, gene family expansion and contraction were analyzed using the Cafe v4.2.1 software [66].

### 4.6. DNA and RNA Extraction and PCR Amplification 

The DNA and cDNA were prepared as previously reported [12,40], which were used to amplify the monospore population with mating type primer (Mat1-1F/R CATGTCACTYCGYCCRGTTTA/CCACATRGCTTTCCTCTTCTC, Mat1-2F/R AACAGATGCTSGAAGAAGC/CTTATAYCCAGGATGCTTTAC) or used to confirm the intron boundaries of mitochondrial genome or the accuracy of sequence assembling results. The amplification system (20 μL) consisted of 0.3 μL of 4× dNTPmix, 2 μL 10× PCR buffer, 10 nM each of primer, 50 ng DNA template, and 1.5 U of rTaq (Takara, Minamikusatsu, Japan), under the conditions of 95 °C for 3 min, followed by 35 cycles of 95 °C for 30 s, 60 °C for 30 s and 72 °C for 60 s. The amplified products were detected by 1.5% agarose gel electrophoresis and sequenced by pyrosequencing technology.

## 5. Conclusions

In this paper, the genome of *M. crassipes* was deeply sequenced and analyzed, and a total of 23 scaffolds were obtained at the subchromosome-scale. The entire genome had a length of 56.7Mb, accounting for 96.7% of the estimated size, and the GC content was 47.3%, containing 5.90% of transposon elements and 9.90% of non-characteristic repeats. Genome integrity assessment showed that 98.62% of the conserved genes in fungi were predicted, indicating that we obtained a virtually complete and fine nuclear genome of *M. crassipes*. Genomic annotation of mating type loci and amplification of mating genes revealed *M. crassipes* was a heterothallic fungus. Importantly, relying on the PacBio long fragment sequencing data, we have also obtained the complete mitochondrial genome physical map of *M. crassipes*, which is the largest mitochondrial genome in fungi so far, nearly twice of that ever reported. One of the main reasons for the enlargement of the mitochondrial genome size was 51 introns, with 19 of them being longer than 5 kb, and their total length being 285,118 bp, accounting for 53.67% of the mitochondrial genome. In addition to conserved mitochondrial genes, 412 ncORFs were identified, with 221 of them being distributed in 51 introns. The total length of these ncORFs was 216,906 bp, accounting for 40.83% of the mitochondrial genome, so that the gene content of the mitochondrial genome was quite dense, with an average of 8.73 genes per 10 K sequence, which was 4.3 times the density of the nuclear genes in *M. crassipes*. Meanwhile, a large number of mitochondrial repetitive sequences were identified in *M. crassipes*, with a total length of 272,544 bp, accounting for 51.31% of the mitochondrial genome. These special characteristics of the nuclear genome and mitochondrial genome of *M. crassipes* could bring novel insights into the understanding of the mysterious yellow morel species.

## Figures and Tables

**Table 1:** Genomic characteristics of *M. crassipes.*

Description	Characteristics
the genome scaffold number (n)	23
the genome contig number (n)	97
the longest length (bp)	7,341,407
the shortest length (bp)	544,727
the genome size estimate (bp)	58,642,780
the genome scaffold size (bp)	56,756,124
the genome contig size (bp)	56,627,527
the rate of N	0.0023
the rate of GC	0.4734
the scaffold N50 (bp)	2,999,225
the contig N50 (bp)	814,084
the scaffold N90 (bp)	1,236,361
the contig N90 (bp)	363,112
the BUSCO genome integrity (%)	98.62

## Supplementary Materials

Supplementary materials can be found at: https://www.mdpi.com/1422-0067/21/2/483/s1.
