# Supplementary figures and images for: Subchromosome-Scale Nuclear and Complete Mitochondrial Genome Characteristics of Morchella crassipes
Source: Int J Mol Sci. 2020 Jan 12;21(2):483. doi: 10.3390/ijms21020483 (PMC7014384; doi:10.3390/ijms21020483)

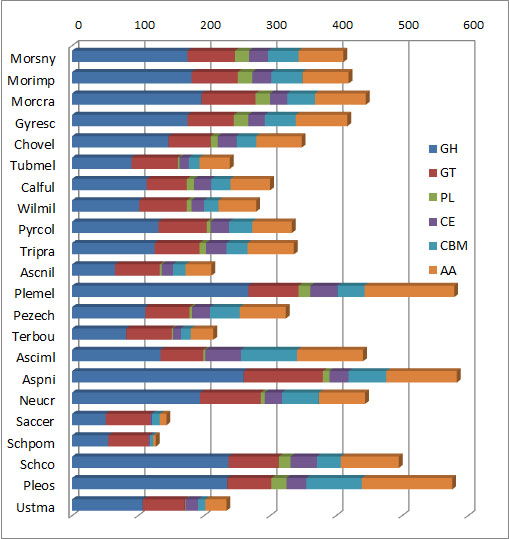

Supplement: Supplementary file 1 [file ijms-21-00483-s001.zip › ijms-633159-SI/Figure S1 CAZYme information of genome╕▒▒╛.jpg]

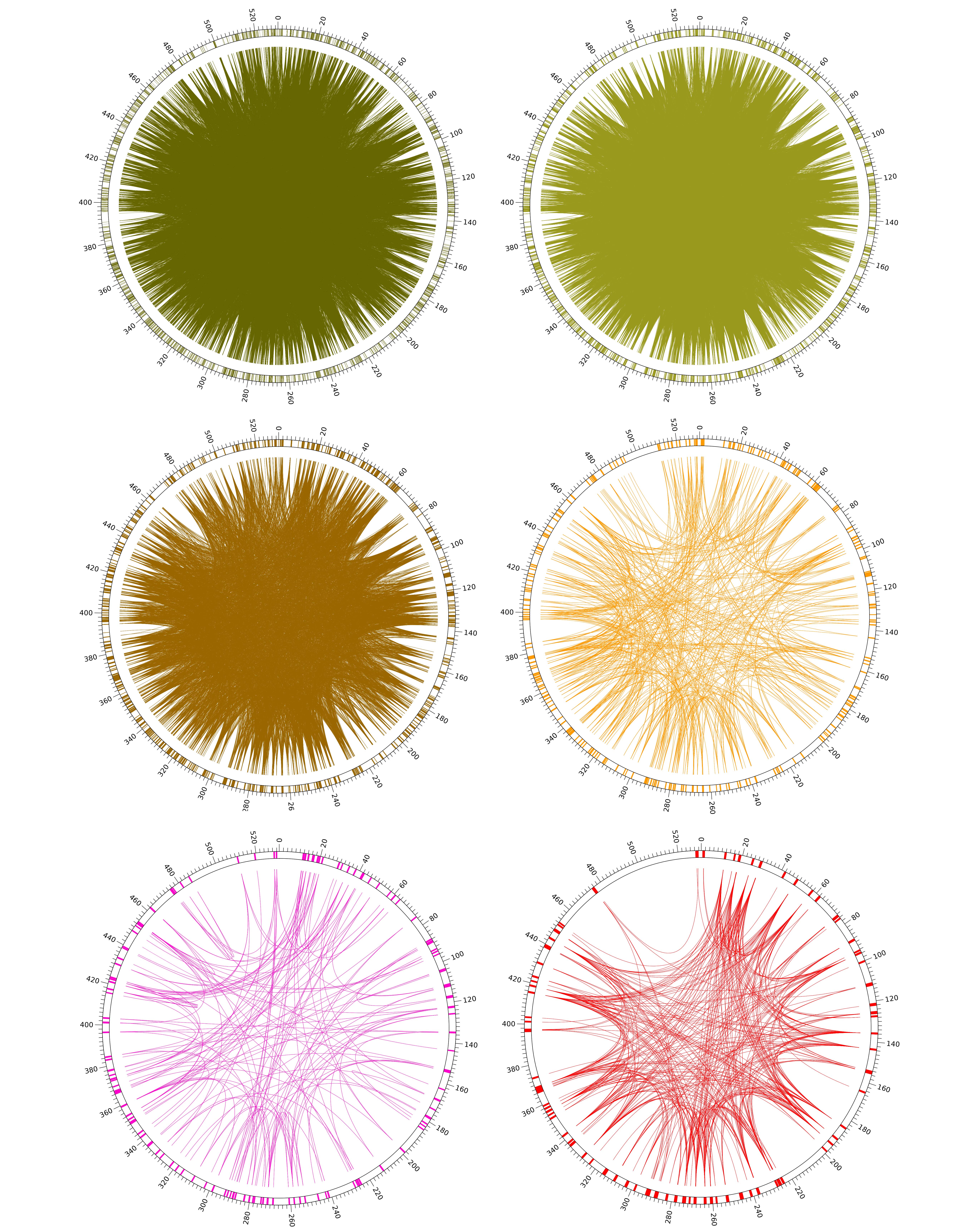

Supplement: Supplementary file 1 [file ijms-21-00483-s001.zip › ijms-633159-SI/Figure S3 Mitochondrial genome different repeat size repeat to self.jpg]
